# City Hospitals as Casualty Stations

**Published:** 1920-02-14

**Authors:** 


### The Hospital

The Workers' Newspaper of Administrative Medicine and Institutional Life, Administration, National Insurance and Health.

No. 1758, Vol. LXVII. SATURDAY, FEBRUARY 14, 1920. PRICE TWOPENCE

### CITY HOSPITALS AS CASUALTY STATIONS.

All who are interested in pressing voluntary hospital problems should read, if they have not already done so, the articles by Mr. E. W. Morris, House Governor of the London Hospital, which appeared in the *Daily Telegraph* on February 2 and 3. These able papers, under the title of "Treatment of the Sick," contain little that is new to the student of hospital affairs, but they marshal the facts necessary to a clear conception of voluntary hospital insufficiencies, and bring home the constant uneconomical use of our all too small accommodation for the reception of the sick, in such a manner that the thesis cannot fail to be consulted in any serious consideration of the problems.

We need not dwell upon the fact that we have too few beds to meet the needs of the population. The outstanding reason for this shortage is that it is now realised that a bed can be more economically used for a patient in the early and possible curable stage of a disease than for one who has become chronic or is at the last gasp. That the modern hospital, with its elaborate and expensive diagnostic organisation, is more suited, and more valuable, to the one than to the other goes without saying. Yet for all that, there is a point when the modern voluntary hospital in its relation to these curable or improvable cases and to cases of accident and emergency, ceases to become economical. This is when these cases reach a stage where they block the way of other curable or improvable cases, and are retained at the hospital solely because, although they should move on, there is no place to move to where treatment can be continued.

The ideal arrangement for getting over these difficulties suggested by Mr. Morris may be stated as follows:—(1) Incurable patients, or those where the small amount of improvement possible does not justify the use of a well-equipped voluntary hospital, should go to the infirmaries; (2) The hospitals should be for the diagnosis and early treatment of curable and improvable cases; (3) These cases, after a short stay in the hospital, but one sufficient for the main purpose, should then proceed to a hut-annexe in the neighbouring country district; (4) Such patients as require it should finally pass on to a convalescent home; (5) A "follow-through" system, with the aid of the general practitioner, watches the case until it is quite sure that all trace of the original trouble has been lost, or the patient is no longer a suitable subject for the hospital system.

Such, briefly, is the plan. As we have said, there is nothing new about it, but Mr. Morris presents well-known facts in such a way that his readers must feel that reformation is needed, is possible, and is comparatively easy. Moreover, the admirable system of the Army Medical Department has brought home to us the possibilities of the casualty station, to them essential and to our peace establishment desirable, and what can be done by means of adequate transport facilities and efficient organisation to prevent blocking of the free movement of patients.

Of course, any system must have disadvantages, and the chief one attaching to the casualty station plan lies in the fact that the surgeon who operates, and who begins a definite course of treatment at the station, does not see the continuation and completion of the treatment and is ignorant as to whether his original intentions are ever carried out. This disadvantage, Mr. Morris thinks, can, with further experience, be obviated. No doubt he has in mind a perfected form of travelling case papers which, while not binding the next responsible medical officer, will at least clearly show the intentions, opinions, and wishes of the physician or surgeon originally in charge of the case. In the hurry and rush of war conditions this was not always possible. With the slower processes of healing in times of peace much more can be done in this direction.

Again, how is the present plan of a voluntary medical staff affected and what becomes of it? Growing complexity in the study of and treatment of disease necessitates some modifications in the system by which hospitals are staffed. There was a time when the hours given by a voluntary staff to their hospital work sufficed, but the demand of the day is for something more, and periodic visits of two or three hours' duration are insufficient for what a hospital has in future to do. To quote Mr. Morris: "I do not say there will be no room for some voluntary service. There will. But there will be much more room for workers 'whose centre of gravity is the hospital,' and not in private practice. The hospital and the work of the hospital must be the first thing in the man's life, measured in terms both of interest and time, and not the second."

Mr. Morris does not deal with the teaching side of the question, which is one of difficulty and as full of complexity as the other aspects. At the stage he has reached the student sees on the medical side but mild forms of disease, and he sees them during only a very short part of their progress. Possibly he may be able to visit the infirmaries and the country annexes. Moreover, the partial disappearance of the voluntary staff clearly affects the student, and one loss amongst others is that of teaching based upon current experience in the world outside the hospital. These are matters for consideration.

From the point of view of economics, under the new plan the cost per occupied bed ceases to be a marking on the financial barometer and its place is taken by the cost per patient. As we have so often said, and as Mr. Morris in effect says now, the cost of the bed is of little interest, and tells one nothing, while we are ignorant of the amount of solid good to the patient, to the advance of medical and surgical science, and ultimately to the health of the nation, that proceeds from its use.

